# Bilirubin—A Possible Prognostic Mortality Marker for Patients with ECLS

**DOI:** 10.3390/jcm9061727

**Published:** 2020-06-03

**Authors:** Sebastian Bunte, Roland Walz, Julia Merkel, Carolin Torregroza, Sebastian Roth, Giovanna Lurati Buse, Hannan Dalyanoglu, Payam Akhyari, Artur Lichtenberg, Markus W. Hollmann, Hug Aubin, Ragnar Huhn

**Affiliations:** 1 Department of Anesthesiology, University Hospital Duesseldorf, Moorenstr. 5, 40225 Duesseldorf, Germany; buntesebastian@gmail.com (S.B.); julia.merkel@hhu.de (J.M.); carolin.torregroza@med.uni-duesseldorf.de (C.T.); sebastian.roth@med.uni-duesseldorf.de (S.R.); giovanna.luratibuse@med.uni-duesseldorf.de (G.L.B.); ragnar.huhn@med.uni-duesseldorf.de (R.H.); 2 Department of Anesthesiology, Amsterdam University Medical Center (AUMC), Meibergdreef 9, 1100 DD Amsterdam, The Netherlands; m.w.hollmann@amsterdamumc.nl; 3 Department of Cardiovascular Surgery, University Hospital Duesseldorf, Moorenstr. 5, 40225 Duesseldorf, Germany; hannan.dalyanoglu@med.uni-duesseldorf.de (H.D.); payam.akhyari@med.uni-duesseldorf.de (P.A.); artur.lichtenberg@med.uni-duesseldorf.de (A.L.); hug.aubin@med.uni-duesseldorf.de (H.A.)

**Keywords:** bilirubin, ECLS, prognosis, liver function, mortality

## Abstract

Extracorporeal life support (ECLS) is a promising therapeutic option for patients with refractory cardiogenic shock. However, as the mortality rate still remains high, there is a need for early outcome parameters reflecting therapy success or futility. Therefore, we investigated whether liver enzyme levels could serve as prognostic mortality markers for patients with ECLS. The present study is a retrospective single-center cohort study. Adult patients >18 years of age who received ECLS therapy between 2011 and 2018 were included. Bilirubin, glutamic-oxaloacetic transaminase (GOT), and glutamic-pyruvic-transaminase (GPT) serum levels were analyzed at day 5 after the start of the ECLS therapy. The primary endpoint of this study was all-cause in-hospital mortality. A total of 438 patients received ECLS during the observation period. Based on the inclusion criteria, 298 patients were selected for the statistical analysis. The overall mortality rate was 42.6% (*n* = 127). The area under the curve (AUC) in the receiver operating characteristic curve (ROC) for bilirubin on day 5 was 0.72 (95% confidence interval (CI): 0.66–0.78). Cox regression with multivariable adjustment revealed a significant association between bilirubin on day 5 and mortality, with a hazard ratio (HR) of 2.24 (95% CI: 1.53–3.30). Based on the results of this study, an increase in serum bilirubin on day 5 of ECLS therapy correlates independently with mortality.

## 1. Introduction

Coronary heart disease remains the number one cause of death in the US. Depending on its severity, it can lead to life-threatening complications such as malignant arrhythmias, cardiogenic shock, or sudden cardiac arrest. According to current data from the American Heart Association (AHA), one out of eight Americans (13.8%) dies from sudden cardiac arrest [1]. Although immediate cardiopulmonary resuscitation (CPR) with immediate defibrillation is crucial to patient survival, the outcome after sudden cardiac arrest is poor and decreases dramatically with the increasing duration of CPR [2,3]. In case of prolonged cardiopulmonary resuscitation, extracorporeal life support (ECLS) can be a therapeutic option for restoring the blood circulation [4,5,6].

Since its first description over 40 years ago, ECLS has become an important tool for managing critically ill patients. Cardiogenic shock in adults has become the most common indication for ECLS therapy, with a reported survival until hospital discharge of up to 42% [7]. Most recent data indicate that ECLS can lead to survival benefits compared to classic CPR in patients with cardiogenic shock or cardiac arrest [8]. However, though promising, the mortality rate when using ECLS is still high. Known complications of ECLS are lethal bleeding in up to 40% of the patients, systemic infections, sepsis, septic shock, as well as acute kidney injuries requiring renal replacement therapy [9,10]. In addition to these complications, liver function plays a crucial role for the outcome of patients treated with ECLS [11]. Acute liver dysfunction and hypoxic liver injury are life-threatening events associated with a mortality rate of up to 80% [12,13]. Some authors tried to identify pre-ECLS factors with prognostic value, e.g., the “Survival after Veno-Arterial Extracorporeal Membrane Oxygenation” (SAVE) score, which may be a tool to predict the survival of patients receiving ECLS for refractory cardiogenic shock [7]. In a retrospective analysis, Freundt et al. showed that bilirubin levels may be related to mortality in patients under ECLS therapy [14]. The authors demonstrated that patients who died under ECLS therapy had significantly higher bilirubin levels than those who survived. Obviously, the initial trauma that leads to initiation of ECLS substantially influences patients’ outcome and may even lead to early therapy futility [10,15,16,17]. With regard to this aspect, it seems even more interesting to evaluate the prognostic value of liver function when this initial damage is excluded as far as possible. In the present study, we therefore investigated whether liver enzyme levels could serve as prognostic markers in patients who survived the first four days of ECLS therapy.

## 2. Materials and Methods

### 2.1. Study Design and Patients

The present study is a retrospective, single-center cohort study, approved by the ethical committee of the Heinrich-Heine-University, Duesseldorf, (reference number 5141R), Germany. Data of all patients older than 18 years of age undergoing ECLS therapy between 2011 and 2018 in our hospital were collected from the institutional database. ECLS therapy and ancillary therapy were performed as previously described [18,19]. All ECLS systems were temporary devices. Only patients who survived the first four days were included into the study, hence excluding patients for whom the initial trauma led to therapy futility and limiting the impact of the index event on liver function and subsequent changes in bilirubin levels. Day 5 was chosen based on local experiences suggesting that this might be the time point of a “steady state” where clinicians often have to decide about prognosis and whether therapy should be continued or not. In a sensitivity analysis, patients presenting without shock liver (defined as a tenfold increase in glutamic-oxaloacetic transaminase (GOT) values compared to the local upper cutoff value (35 U/L)) were excluded. This subgroup analysis was performed to further exclude the influence of the initial damage.

### 2.2. Data Collection

Data including sex, age, duration of ECLS therapy, and blood levels of total bilirubin, GOT and glutamic-pyruvic-transaminase (GPT) were collected at pre-defined time points: day 0 (start of ECLS therapy) and day 5 (after five days of ECLS therapy). If no blood values were available on the corresponding days, results from the adjacent days (cut-off date ± 2 days) were used.

### 2.3. Statistical Analysis

The primary endpoint of this trial was all-cause in-hospital mortality. Categorical data are presented as absolute numbers (percent). Continuous data are presented as mean ± standard deviation (SD) or median (quartile 1, quartile 3, as applicable). Differences between baseline characteristics were calculated by Pearson χ^2^ test, Fisher exact test, Student *t* test, or Mann–Whitney *U* test, as appropriate. The discrimination of bilirubin for mortality was examined employing receiver operating characteristic curve (ROC) and the area under the curve (AUC). The Youden index was calculated to determine the cutoff value for bilirubin. To quantify the independent association between bilirubin and death, Cox regression was performed with pre-defined multivariable adjustment for sex, age, left ventricular assist device/right ventricular assist device (LVAD/RVAD), and continuous renal replacement therapy (CRRT). Cox regression was also performed for GOT and GPT. In a predefined sensitivity analysis, patients with shock liver (defined as GOT >10 × cutoff (= 35 U/L)) were excluded.

## 3. Results

### 3.1. Baseline Characteristics

A total of 438 patients underwent ECLS therapy during the observation period. All patients received ECLS due to severe cardiogenic shock or cardiac arrest. Of these, 298 patients survived the first four days, and their data were available for statistical analysis (see Table 1). To allow generalizability, all indications for ECLS therapy were included. The median age was 59 ± 14 years, 219 (74%) patients were male, 79 (26%) were female. The mean ECLS therapy duration was 9 ± 7 days. With regard to the baseline characteristics, the only significant difference was sex (*p* <0.05). For the sensitivity analysis without shock liver constellation, 220 patients remained.

### 3.2. Laboratory Values and Outcome

**Figure 1:**
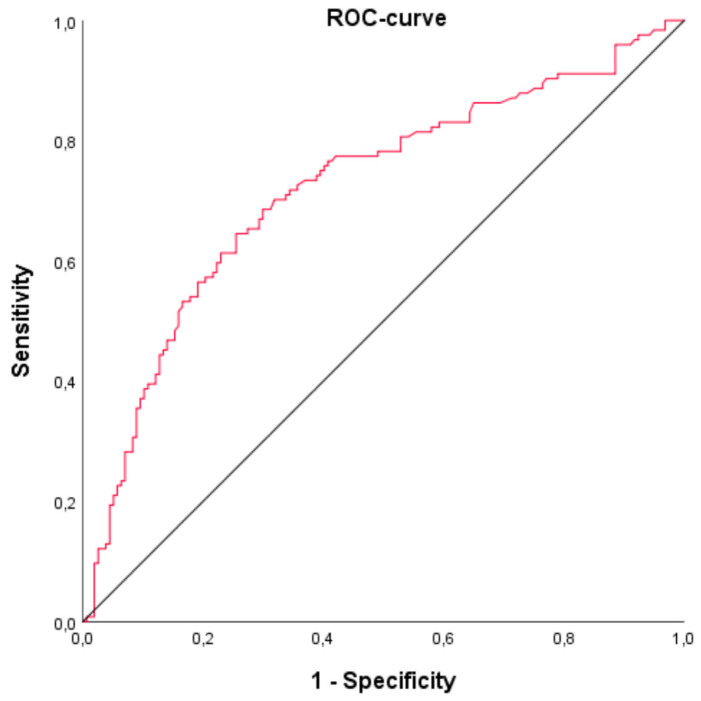
Receiver operating characteristic curve (ROC) and area under the curve (AUC) for bilirubin on day 5 of ECLS therapy as a discriminator for mortality (*n* = 298). AUC = 0.72 (95% confidence interval (CI): 0.66–0.78).

**Figure 2:**
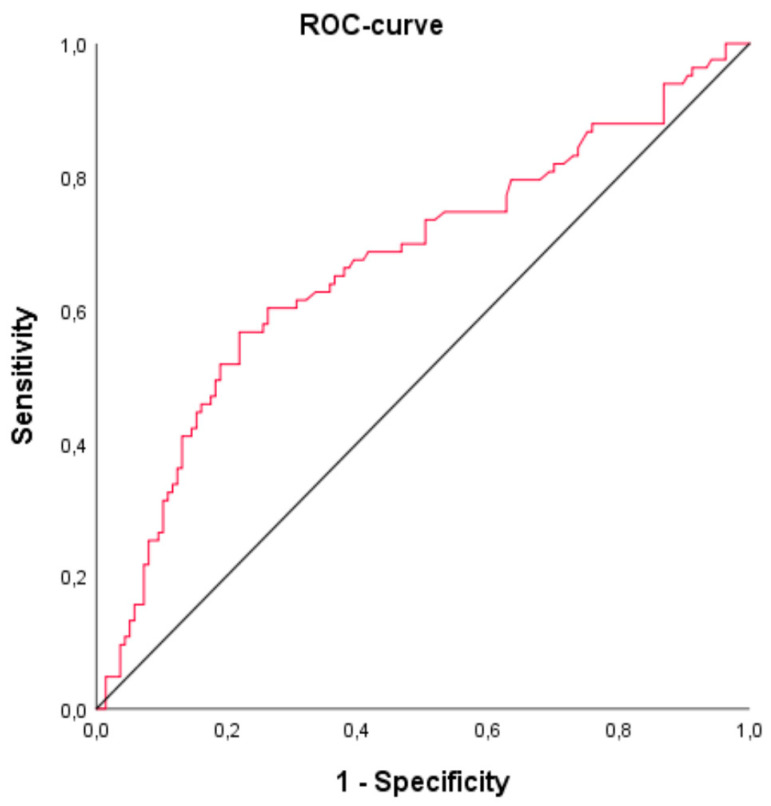
ROC and AUC for bilirubin on day 5 of ECLS therapy as a discriminator for mortality, excluding shock liver patients (*n* = 220). AUC = 0.67 (95% confidence interval (CI): 0.59–0.75).

Laboratory values were followed over a period of five days from the start of the ECLS therapy. In-hospital mortality rate was 42.6% (*n* = 127). The mean bilirubin level at the beginning of ECLS was 2.04 ± 2.73 mg/dL, and the mean bilirubin level on day five was 5.14 ± 12.01 mg/dL. The AUC from the ROC for bilirubin on day five was 0.72 (95% confidence interval (CI): 0.66–0.78) (see Figure 1). The AUC for the trend (i.e., the absolute difference) between bilirubin at the start and on day 5 was 0.70 (95% CI 0.64–0.77). ROC for patients without shock liver demonstrated an AUC of 0.67 (95% confidence interval (CI): 0.59–0.75) (see Figure 2).

**Figure 3:**
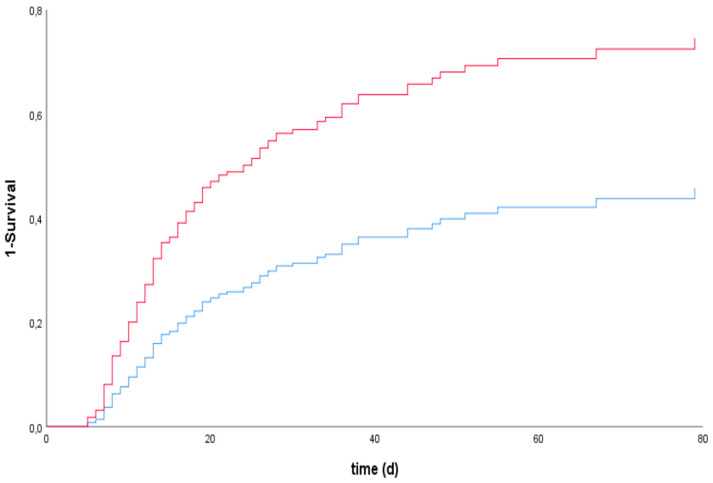
Cox proportional hazards model for investigating the association between the survival time of patients undergoing ECLS therapy and bilirubin levels on day 5 if bilirubin was >2.23 mg/dL (red line) or <2.23 mg/dL (blue line) (cutoff value as determined by the Youden index), including a predefined multivariable adjustment.

**Figure 4:**
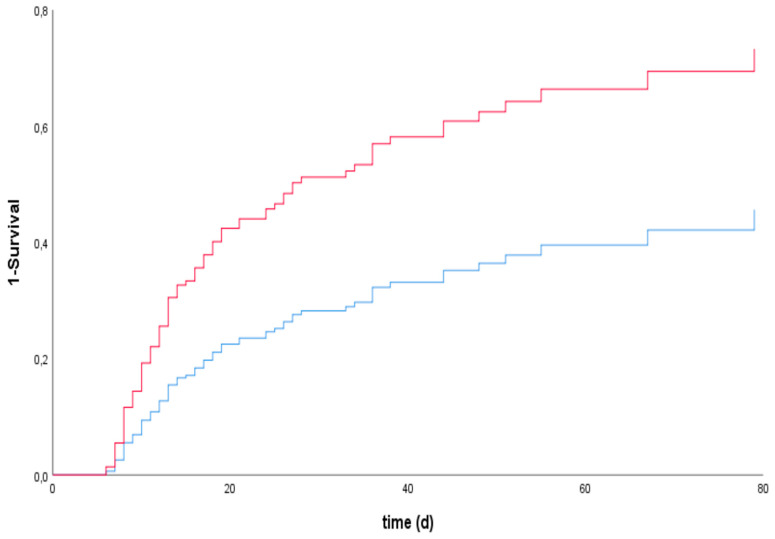
Cox proportional hazards model for investigating the association between the survival time of patients undergoing ECLS therapy with the exclusion of shock liver patients and bilirubin levels on day 5 if bilirubin was >2.23 mg/dL (red line) or <2.23 mg/dL (blue line) (cutoff- alue as determined by the Youden index), including a predefined multivariable adjustment.

The Youden index showed a cutoff for bilirubin on day 5 of 2.23 mg/dl, with a sensitivity of 0.70. Cox regression with multivariable adjustment revealed a significant association between bilirubin on day 5 and mortality, with a hazard ratio (HR) of 2.24 (95% CI: 1.53–3.30) (see Table 2 and Figure 3). In the sensitivity analysis without shock liver patients, this association was still significant (HR 2.08 (95% CI: 1.33–3.26) (see Table 2 and Figure 4). Cox regression for GOT and GPT showed a significant association between GOT on day 5 and mortality, but not between GPT on day 5 and mortality, with HR of 1.87 (95% CI: 1.23–2.83) for GOT and 1.41 (95% CI: 0.94–2.10) for GPT (see Table 3).

## 4. Discussion

ECLS therapy is a promising treatment for life support of cardiogenic shock patients. However, the method itself also involves many potential lethal complications such as acute bleeding, futile intracerebral stroke, or kidney failure [9,10]. In addition to these factors, acute liver failure is a serious complication of ECLS therapy, with potential survival implications [14,15,16,17]. In recent years, some studies tried to identify pre-ECLS factors that may predict survival in these patients. In 2015, Schmidt and co-workers created the SAVE score and concluded that this score may be a tool to predict survival in patients receiving ECLS for refractory cardiogenic shock [20]. In everyday clinical practice, however, there is no reliable prognostic marker of mortality for patients that survived the first few days of therapy. This time point may be even more important, for example, to decide whether therapy should be limited or not. The results of our current study suggest that bilirubin measured on day 5 of ECLS might serve as a prognostic marker in this context and thus could help guide therapy.

In general, liver function is important for the survival of patients undergoing ECLS, and an elevated baseline bilirubin inversely correlates with long-term survival [11]. In 2016, Roth et al. confirmed the latter results and suggested that bilirubin and alkaline phosphatase are predictors of 30-day and long-term mortality following ECLS. Strikingly, the relevance of the bilirubin values during the course of ECLS remained unclear. Obviously, the initial trauma that leads to the initiation of ECLS therapy influences patients’ outcome and may even lead to early therapy futility. Therefore, it seems even more interesting to evaluate the prognostic value of bilirubin when this initial damage is excluded as far as possible. Hence, this is the first study to investigate the potential of bilirubin as a prognostic marker in patients who survived the first few days of ECLS therapy.

Freundt et al. were the first to look at the time course of bilirubin values with regard to mortality [14]. Patients that survived after explantation of the device had a regression of their bilirubin levels within two days, whereas patients that died showed a further bilirubin increase. The authors concluded that a decrease in bilirubin levels over time might be a predictor of successful weaning from ECLS in hemodynamically stable patients [14].

Those data support the idea that bilirubin values during ECLS therapy may be more relevant with regard to prognostic information than baseline values and may support an early recognition of problems as well as the optimization of the therapy. Worth mentioning, the study protocol in the latter study differed significantly from ours. Freundt et al. included all patients that received ECLS therapy and followed them over a period of six days and at least two days after explantation. The authors stated that the mean ECLS duration was three days [14]. In our current study, patients who died within the first four days of ECLS therapy were excluded from the study, and the mean ECLS duration was nine days. The admission of initially deceased patients as well as the short ECLS duration make it very difficult to distinguish whether the initial trauma or the ECLS therapy was the cause of the measured effects. Roedl et al. showed that the original trauma has a major effect on the liver function of patients [16]. The authors demonstrated that a trauma such as cardiac arrest can trigger an impairment of the liver function such as hypoxic liver injury (HLI) [16]. Out of 1068 patients with cardiac arrest after CPR, 21% developed HLI [16]. Iesu et al. investigated the occurrence of acute liver failure (ALF) after cardiac arrest. In their study, 56% of patients with return of spontaneous circulation (ROSC) developed acute liver failure that significantly affected mortality. Iseu et al. also showed that ALF occurred within the first three days after the initial event due to the elevation of bilirubin [15].

Based on the results of Roedl et al. and Iseu et al., we did our best to avoid a possible influence of the initial event (e.g., cardiogenic shock) on liver function. Therefore, all patients that did not survive at least four days after ECLS implantation in our study were excluded. When comparing our data with those of Freundt et al., it is noteworthy that in our study no significant difference in the initial bilirubin values between the groups existed. However, during ECLS therapy, a significant increase in bilirubin was detected. Even more, as determined by the Youden index, our data show that bilirubin levels with a cutoff of 2.23 mg/dL and a sensitivity of 0.7 on day 5 of ECLS therapy correlated with increased mortality (see Figure 3). Although this cutoff represents the point with the highest sensitivity and specificity according to the ROC analysis, it has to be taken into account that bilirubin levels were higher in most patients (mean on day five, 5.14 ± 12.01) and not every level of bilirubin slightly higher than normal is correlated with high mortality. However, this cutoff may serve as a “warning signal” for clinicians treating ECLS patients.

To further minimize the involvement of the initial trauma in liver function for our results, we performed a subgroup analysis in which we excluded all patients with an initial shock liver, defined as a tenfold increase in GOT values compared to the upper cutoff value (35 U/L). The association of bilirubin and mortality, which was seen before, was significant also in this case (see Table 2 and Figure 4).

However, given the fact that we did a retrospective analysis, we cannot rule out an influence of the initial event on bilirubin levels. In order to be able to make a definite statement, a prospective, randomized study should be carried out. It also has to be mentioned that we showed no significant difference between GPT and mortality (see Table 3), indicating that other underlying mechanisms than liver injury may have led to increased bilirubin values, e.g., hemolysis. Nonetheless, combined with previously published work, our results suggest that bilirubin levels might be a prognostic marker for mortality in patients with ongoing ECLS therapy, useful to prevent or recognize therapy-related problems and to optimize treatment.

### Limitations

Our study has the following limitations. First, an important limitation is the retrospective design. Second, this study is a single-center study, and the therapy of ECLS patients may be totally different in other centers so that we cannot generalize our findings. Third, we only looked at bilirubin measurements at baseline and on day 5 of ECLS therapy. We did this to exclude the initial trauma as far as possible and to define a time point that might be clinically relevant with regard to prognostication and optimization of therapy. However, more bilirubin values would be useful to evaluate the importance of its changes over time. These values were not included in the analysis because of a relevant amount of missing data which is related to the retrospective data collection. Fourth, there was a significant difference depending on sex in the baseline characteristics of our cohort which may also influence generalization. Fifth, our decision to focus on day 5 was based on local experience, and there is no scientific reference supporting this choice. Sixth, our database did not include more variables to be considered in the multivariate analysis.

## 5. Conclusions

In summary, our results suggest that serum bilirubin may act as a prognostic marker for mortality in patients undergoing ECLS therapy who survived the first few days of therapy. In those patients, bilirubin may be used for an early recognition of problems and thus could be seen as a useful indicator for early optimization of ECLS therapy or to guide decision-making.

## Figures and Tables

**Table 1:** Patient characteristics.

	All Patients	Non-Survival	Survival	*p*-Value
Number of Patients (*n*) (%)	298	133 (45%)	165 (55%)	ns
Age (Years)	59 ± 14	61 ± 14	56 ± 14	ns
Male (*n*) (%)	219	100 (46%)	119 (54%)	ns
Female (*n*) (%)	79	33 (42%)	46 (58%)	ns
Days of ECLS	9 ± 7	11 ± 7	8 ± 7	ns

Data are presented as mean ± SD or as absolute numbers (percent). ECLS = extracorporeal life support.

**Table 2:** Multivariate analysis for bilirubin with and without shock liver patients.

	Hazard Ratio	Lower 95% CI	Upper 95% CI	*p*-Value
Bilirubin on day 5 including shock liver patients (*n* = 298)				
Bilirubin	2.243	1.525	3.297	<0.0001
Age	1.010	0.996	1.024	0.153
Sex	1.119	0.743	1.684	0.590
LVAD	0.433	0.235	0.795	0.007
RVAD	1.268	0.630	2.551	0.506
CRRT	1.758	1.142	2.707	0.010
Bilirubin on day 5 excluding shock liver patients (*n* = 220)				
Bilirubin	2.080	1.328	3.256	0.001
Age	1.013	0.996	1.031	0.136
Sex	1.243	0.753	2.051	0.395
LVAD	0.334	0.154	0.721	0.005
RVAD	2.000	0.822	4.865	0.127
CRRT	1.640	1.005	2.677	0.048

LVAD = left ventricular assist device; RVAD = right ventricular assist device; CRRT = continuous renal replacement therapy.

**Table 3:** Multivariate analysis for glutamic-oxaloacetic transaminase (GOT) and glutamic-pyruvic-transaminase (GPT).

	Hazard Ratio	Lower 95% CI	Upper 95% CI	*p*-Value
GOT on day 5				
GOT	1.868	1.233	2.830	0.003
Age	1.011	0.998	1.025	0.088
Sex	1.223	0.816	1.835	0.330
LVAD	0.460	0.249	0.848	0.013
RVAD	1.387	0.688	2.793	0.360
CRRT	1.667	1.070	2.599	0.024
GPT on day 5				
GPT	1.406	0.941	2.100	0.097
Age	1.017	1.002	1.031	0.021
Sex	1.222	0.805	1.854	0.346
LVAD	0.518	0.270	0.993	0.047
RVAD	1.411	0.686	2.901	0.350
CRRT	1.933	1.209	3.093	0.006
